# A clinical analysis of *Candida tropicalis* bloodstream infections associated with hematological diseases, and antifungal susceptibility: a retrospective survey

**DOI:** 10.3389/fmicb.2023.1092175

**Published:** 2023-07-14

**Authors:** Beibei Yang, Zhenbin Wei, Meiqing Wu, Yongrong Lai, Weihua Zhao

**Affiliations:** Department of Hematology, The First Affiliated Hospital of Guangxi Medical University, Nanning, China

**Keywords:** *Candida tropicalis*, bloodstream infections, hematological disorders, prognosis, antifungal susceptibility

## Abstract

**Summary objective:**

To assess the clinical features and outcomes of hematological disease patients with *Candida tropicalis* bloodstream infections and determine the antifungal susceptibility of *C. tropicalis*.

**Methods:**

This is a retrospective, single-center, observational study conducted in the Department of Hematology at The First Affiliated Hospital of Guangxi Medical University from January 2013 to December 2021. A total of 26 hematological disease patients with *C. tropicalis* bloodstream infections were enrolled, and their clinical features, treatment plans, and prognoses were assessed. Univariate analysis was performed by Kaplan–Meier analysis and multivariate analysis was conducted using a Cox regression model. The antifungal susceptibility of *C. tropicalis* was determined from patient blood cultures.

**Results:**

The patients had a mean age of 35 years (range: 10–65 years), 50% were male (13/26) and 88.5% had hematologic malignancies (23/26) while the remaining three patients included two cases of severe aplastic anemia and one case of β-thalassemia. All patients had neutropenia. Seven patients were initially given azole alone (26.9%), five of whom failed treatment and died (71.4%). Fifteen patients were treated with echinocandin (57.7%), three of whom failed treatment and died (20.0%), and eight patients were treated with amphotericin B (30.8%), two of whom failed treatment and died (25.0%). The total and attributable mortality rates were 42.3 and 34.6%, respectively. Univariate analysis showed that there are six risk factors for attributable deaths among hematological disease patients with *C. tropicalis* blood infections. These risk factors included septic shock, Pitt bacteremia scores ≥4, procalcitonin levels ≥10 ng/mL, positive plasma (1,3)- β-D glucan assay, serum albumin levels <30.0 g/L, time from fever to antifungal treatment initiation ≥5 days and time between neutropenia and antifungal treatment ≥10 days. Moreover, skin and mucosal infections and a treatment schedule that included amphotericin B and drug combinations are protective factors for attributable deaths. Multivariate analysis showed that septic shock (*p* = 0.006) was an independent risk factor for attributable death. All isolates were sensitive to flucytosine and amphotericin B. The intermediate or resistance of *C. tropicalis* to fluconazole, itraconazole and voriconazole were 41.7, 50, and 41.7%, respectively.

**Conclusion:**

Hematological disease patients with *C. tropicalis* bloodstream infections had a high mortality rate, and early antifungal therapy significantly reduced mortality. *Candida tropicalis* was highly resistant to azole drugs and sensitive to flucytosine and amphotericin B. According to our study, the preferred agent is amphotericin B and drug combinations should be considered for severe infections.

## 1. Introduction

The incidence of Candida bloodstream infections (CBI) has increased dramatically in recent decades and Candida is now the most common etiology of fungal sepsis (Lamoth et al., 2018; Arastehfar et al., 2021). In the United States, Candida is the fourth most common cause of nosocomial bloodstream infections (BSI; Wisplinghoff et al., 2004). In Europe, Candida is the third most common etiology of fungal sepsis and is associated with a 37% 30-day mortality rate (Koehler et al., 2019). The Chinese Consensus on the Diagnosis and Management of Adult Candidiasis has observed a substantial increase in the incidence of invasive fungal disease (IFD), of which Candida is the primary cause (Gui-Qiang, 2020). *Candida* sepsis is associated with prolonged hospitalization times and increased costs, burdening both patients and the healthcare system (Hassan et al., 2009; Bloos et al., 2013; Heimann et al., 2015).

In Asian countries, non-albicans *Candida* spp. (NAC) is more prevalent than *Candida albicans* (Lortholary et al., 2017; Zhang et al., 2020; El Zakhem et al., 2021). Hematologic disorders are significant risk factors for Candida infection due to several factors including neutropenia, frequent use of immunomodulatory agents, prolonged use of broad-spectrum antimicrobials, central vascular catheters (CVC), chemotherapy and hematopoietic stem cell transplantation (HSCT; Kashefi et al., 2021). The highest percentage of *Candida tropicalis* among blood isolates was found in haemato-oncology wards (Tan et al., 2015). This fungus is associated with the highest rates of sepsis and the poorest prognosis of any *Candida* spp. (Ko et al., 2019). The emergence of antifungal-resistant *C. tropicalis* has also been widely reported in recent years (Zuza-Alves et al., 2017). Multiple studies have shown that almost half of Candidemia patients infected with *C. tropicalis* are azole-resistant (Chong et al., 2012; Chen et al., 2019; Arastehfar et al., 2020a,b).

The study of *C. tropicalis* bloodstream infections (CTBI) with hematological diseases still needs further exploration. This study describes the clinical features of CTBI in a hematology ward, evaluates the risk factors associated with mortality, and analyzes the antifungal susceptibility of this pathogen. Understanding CTBI-associated hematologic diseases are critical for the development of effective prevention and treatment options.

## 2. Methods

### 2.1. Study design

This is a retrospective, single-center, observational study conducted in the Department of Hematology at The First Affiliated Hospital of Guangxi Medical University. All patients who were diagnosed with a CTBI-associated hematological disease between January 2013 and December 2021 were included in the study. Clinical information was collected from medical records and included underlying disease type, therapeutic methods, treatment conditions, clinical symptoms, signs, laboratory indices, neutropenia, immunomodulatory agents, broad-spectrum antimicrobials, CVC used, antifungal therapy, therapeutic outcomes, and *C. tropicalis* antifungal sensitivity.

### 2.2. Inclusion and exclusion criteria

All patients with a CTBI-associated hematological disease that included at least one positive *C. tropicalis* blood culture and the presence of clinical signs and symptoms outlined by the Chinese Society of Hematology (Xiaojun, 2020) and the Infectious Diseases Society of America (IDSA; Pappas et al., 2015) were included in the study. Patients who were diagnosed with the non-blood system-related disease or who had a *C. tropicalis*-positive blood culture with symptoms that improved without treatment were excluded.

### 2.3. The definition of antifungal resistance and susceptibility

According to the NCCLS M27-A standard, infection was considered to be antifungal resistant if cultured *Candida* spp. isolates exhibited a voriconazole minimal inhibitory concentration (MIC) above 2 mg/L, fluconazole above 64 mg/L, itraconazole above 1 mg/L, amphotericin B above 32 mg/L and flucytosine above 16 mg/L. Conversely, it was considered to be antifungal susceptibility if voriconazole MIC below 0.125 mg/L, fluconazole below 8 mg/L, itraconazole below 0.125 mg/L, amphotericin and flucytosine below 4 mg/L.

### 2.4. Patient follow-up

Patients were followed up by telephone and through both inpatient and outpatient visits. The starting point of follow-up was the time of the first positive blood culture and the end point was death. The follow-up period was the time from etiological diagnosis to death, the end of the study period, or loss to follow-up. The mean follow-up time to the end of the study period on January 31, 2022, was 176 days (range: 4–708 days).

### 2.5. Blood culture and drug sensitivity

Blood specimen was obtained and cultured before or at the peak of each patient’s fever. The culture and isolation of strains were conducted according to the National Clinical Test Regulations of Operation, using Bactec FX blood culture instruments and matching blood culture bottles (Becton, Dickinson, and company). Strain identification and drug sensitivity tests were conducted using the Phoenix100 fully automatic analyzer. The results were determined using the 2017 Clinical Laboratory Standards Institute (CLSI) standards.

### 2.6. Statistical analyses

SPSS 20 was used for data analysis. Means and extremums were used to compute continuous variables such as age. Frequency and percentage were used to determine qualitative variables including underlying disease type and clinical signs and symptoms. Univariate analysis was performed using the Kaplan–Meier test and multivariate analysis was conducted using the Cox regression. *p* < 0.05 were considered statistically significant.

### 2.7. Ethics approval

All procedures were performed following relevant guidelines. This manuscript has been approved by the ethics committee of The First Affiliated Hospital of Guangxi Medical University. Oral informed consent was obtained from all patients included in the study.

## 3. Results

### 3.1. Clinical features

A total of 26 cases were enrolled by the Department of Hematology. The mean age was 35 years (range: 10–65 years), and 50% of the patients were male (13/26). A total of 23 cases had hematologic malignancies (88.5%) and the specific types are described in Table 1. The three remaining cases had nonmalignant disorders including one case with β-thalassemia (3.8%) and two with severe aplastic anemia (SAA; 7.7%). The patients with hematologic malignancies included six newly diagnosed patients, nine who had achieved complete remission, one who had partial remission, three who had no remission after induction chemotherapy, and four who relapsed. Twenty-two of the hematologic malignancy patients received chemotherapy, one of whom had therapy prior to HSCT. Of the three nonmalignant patients, two had received HSCT, and one SAA patient had received anti-thymocyte globulin (ATG) therapy prior to the current study.

**Table 1:** Characterization of patients with CTBI-associated hematologic diseases.

Observation	CBI	Rate
**Underling disease**		
AML	16	61.5%
ALL	5	19.2%
NHL	1	3.8%
Mixed acute leukemia	2	7.7%
SAA	2	7.7%
β-thalassemia	1	3.8%
**Condition of malignancy**		
Newly diagnosed	6	26.1%
CR	9	39.1%
PR	1	4.3%
Non-remission	3	13.0%
Relapse	4	17.4%
**Predisposing factor**		
Chemotherapy	22	84.6%
HSCT	2	7.7%
Therapy before HSCT	1	3.8%
ATG therapy	1	3.8%
CVC	21	80.8%
Immunomodulatory therapy	3	11.5%
Neutrophil	26	100%
Broad-spectrum antibiotic exposure	26	100%

ALL, acute lymphoblastic leukemia; AML, acute myeloblastic leukemia; ATG therapy, anti-thymocyte globulin; CR, complete remission; CTBI, *C. tropicalis* bloodstream infections; CVC, central vena catheterization; HSCT, hematopoietic stem cell transplantation; NHL, non-Hodgkin lymphoma; PR, partial remission; SAA, severe aplastic anemia.

All patients developed neutropenia, defined as a neutrophil count <0.5 × 10^9^ /L, with an average duration of 19 days (range: 4–61). All patients had broad-spectrum antibiotic exposure, 21 had CVC, and three received immunomodulatory agents.

Digestive symptoms, such as abdominal pain, vomiting, diarrhea, and hematochezia, occurred in 15 patients, and respiratory symptoms, such as cough, sputum, or hemoptysis occurred in 21 patients. All patients had a fever, of whom 14 had temperatures >40°C. A total of 15 patients had skin or mucosal infections, including nine patients with oral infection, five with perianal infection, four with skin plaques, two with pharyngeal infections, one with a local skin infection, and one with a perineum infection. Ten patients experienced septic shock, one of the most serious BSI-associated complications.

### 3.2. Laboratory and pathogen testing

Our study checked for markers of infection in these patients, such as procalcitonin (PCT), hypersensitive C-reactive protein (CRP), galactomannan (GM), and plasma (1,3)- β-D glucan assay (BG). However, not all patients were checked for all markers of infection. According to the clinical dates, 52.6% of patients had a PCT ≥ 0.5 ng/mL (10/19), 21.1% of patients had a PCT ≥ 10 ng/mL (4/19), all patients had a CRP ≥ 10 mg/L, 68.8% had a CRP ≥ 100 mg/L (11/16), 38.9% had a BG ≥ 10 ng/L (7/19) and 66.7% had a GM ≥ 0.5 (10/16). Serum albumin <30.0 g/L was present in 42.3% of patients (11/26) (Table 2).

**Table 2:** Patient clinical symptoms and blood and pathogen testing results.

Observations	CBI	Rate
**Clinical symptoms**		
Respiratory symptoms	21	80.8%
Digestive symptoms	15	57.7%
Fever	26	100%
37.7–39.9°C	12	46.2%
≥40.0°C	14	53.8%
Central nervous system	2	7.7%
Septic shock	10	38.5%
Heart failure	4	15.4%
Skin or mucosal infection	15	57.7%
Oral infection	9	34.6%
Perianal infection	5	19.2%
Pharyngeal infection	2	7.7%
Skin plaque	4	15.4%
Local skin infection	1	3.8%
Perineum infection	1	3.8%
**Pitt Bacteremia Score**		
<4	18	69.2%
≥4	8	30.8%
**Laboratory testing**		
PCT ≥ 10 ng/mL	4/19	21.1%
CRP ≥ 100 mg/L	11/16	68.8%
BG ≥ 10 ng/L	7/18	38.9%
GM ≥ 0.5	10/15	66.7%
**Pathogen testing**		
*Blood culture*		
*C. tropicalis*	26	100%
Co-infected with *C. glabrata*	1	3.8%
Co-infected with *C. albicans*	1	3.8%
Associated with another bacteremia	1	3.8%
*Klebsiella pneumoniae*	1	3.8%
*E. coli*	1	3.8%
*Staphylococcus haemolyticus*	1	3.8%
*E. coli* with *Pseudomonas aeruginosa*	12	42.9%
*E. coli* with *Streptococcus gallolyticus*	9	32.1%
Stool or perianal swab grew Candida	26	100%
Sputum or throat swab grew Candida	1	3.8%

BG, plasma (1,3)- β-D glucan assay; CBI, Candida bloodstream infections; CRP, hypersensitive C-reactive protein; *E. coli*, *Escherichia coli*; GM, galactomannan test; PCT, procalcitonin.

There are two patients who were not only infected with *C. tropicalis* but also infected with other candida (*C. glabrata* and *C. albicans*, respectively). Candida was grown in the stool of 12 patients and from the sputum or throat swabs of eight patients. Blood cultures from six patients were positive for other pathogens (23.1%), including two that were positive for *Klebsiella pneumonia*, one for *E. coli*, one for *Staphylococcus haemolyticus*, one for *E. coli* and *Pseudomonas aeruginosa* and one for *E. coil* and *Streptococcus gallolyticus*.

### 3.3. Treatment and outcomes

CVC was removed from 11 patients during infection (52.4%). Six patients received azole as an antifungal prophylaxis prior to fever (23.1%), 15 received empiric treatment between their fever and the return of blood culture results (57.7%), and seven initially received azole alone at the beginning of fever (26.9%). Each of these patients received a change in the treatment regimen, or the addition of echinocandin, amphotericin B, or flucytosine because the therapy was not effective, however, five patients failed treatment and died (5/7, 71.4%). In addition, 15 patients were treated with echinocandin (57.7%), three of whom failed treatment and died (3/15, 20.0%), and eight were treated with amphotericin B (30.8%), including two Amphotericin B Liposome, two of whom failed treatment and succumbed to disease (2/8, 25.0%).

By the end of the study on January 31, 2022, the median follow-up time was 176 days (4,708), the total antifungal treatment effectiveness was 65.4% (17/26), the total mortality rate was 42.3% (11/26), the attributable mortality rate was 34.6% (9/26), and the 28-day mortality rate was 26.6% (7/26).

### 3.4. Risk factors of CTBI-associated mortality

Univariate analysis showed that there are six risk factors for attributable deaths among hematological disease patients with *C. tropicalis* blood infections. These risk factors included septic shock, Pitt bacteremia scores ≥ 4, PCT ≥ 10 ng/mL, positive BG, serum albumin levels <30.0 g/L, time from fever to antifungal treatment initiation ≥5 days and time between neutropenia and antifungal treatment ≥10 days (*p* < 0.05; Table 3). Moreover, skin or mucosal infections, and a treatment schedule including amphotericin B or drug combinations, such as caspofungin, voriconazole with amphotericin B or caspofungin, flucytosine with amphotericin B, including three Amphotericin B Liposome, were factors associated with favorable outcomes (*p* < 0.05; Table 3).

**Table 3:** Univariate analysis of death-related factors attributable to CTBI.

	Survival group	Death group	*p*	*Χ* ^2^
**Basic complication**				
Hematologic malignancies	15	8	0.829	0.018
Non-CR	8	6	0.255	1.293
HSCT	1	1	0.838	0.042
Chemotherapy	14	8	0.604	0.269
Hospital stay ≥40 days	8	3	0.369	0.806
**Predisposing factor**				
Time of neutropenia ≥20 days	5	5	0.152	2.049
Immunomodulatory agents	1	2	0.142	2.156
CVC	16	5	0.539	0.377
**Clinical symptoms**				
Respiratory symptoms	14	7	0.929	0.008
Digestive symptoms	8	7	0.156	2.010
Fever ≥40°C	8	6	0.454	0.560
Septic shock	2	8	0.000	14.744
Heart failure	2	2	0.569	0.324
Skin or mucosal infection	12	3	0.035	4.437
Hepatomegaly	0	1	0.194	1.683
Splenomegaly	3	1	0.124	2.360
Pitt Bacteremia Score ≥ 4	1	7	0.000	13.368
**Laboratory index**				
PCT ≥ 10 ng/mL	1	3	0.042	4.121
CRP ≥ 100 mg/L	6	5	0.083	3.004
BG ≥ 10 ng/L	2	5	0.009	6.846
GM ≥ 0.5	8	2	0.264	1.246
Serum albumin <30.0 g/L	4	7	0.006	7.420
**Pathogen testing**				
Cultured Candida in stool	6	6	0.113	2.506
Cultured Candida in sputum	6	2	0.561	0.338
Associated with another bacteremia	3	3	0.583	0.302
**Treatment**				
Time from fever to antifungal treatment initiation ≥5 days	3	6	0.011	6.417
Time from neutropenia to antifungal treatment ≥10 days	2	6	0.004	8.271
Removed CVC	8	2	0.678	0.173
Antifungal prophylaxis	5	1	0.242	1.369
Empiric treatment	8	3	0.365	0.820
**Therapeutic schedule**				
Caspofungin	15	8	0.985	0.000
Azoles	11	4	0.346	0.887
Amphotericin B	15	5	0.041	4.186
Flucytosine	9	2	0.084	2.976
Drug combination	16	6	0.028	4.816

BG, plasma (1,3)- β-D glucan assay; CRP, hypersensitive C-reactive protein; CTBI, *C. tropicalis* bloodstream infections; CVC, central vena catheterization; GM, galactomannan test; HSCT, hematopoietic stem cell transplantation; non-CR, including newly diagnosed, partial remission, no remission after induction of chemotherapy, and relapse; PCT, procalcitonin.

Multivariate analysis was performed using the Cox regression model. Findings revealed that septic shock (*p* = 0.006; Table 4) is an independent risk factor for death attributed to CTBI.

**Table 4:** Multivariate analysis of death-related factors attributable to CTBI.

Risk factors	B	*p*	HR	95.0% CI of HR
Septic shock	2.958	0.006	19.257	2.361 ~ 157.056

CTBI, *C. tropicalis* bloodstream infections.

### 3.5. Antifungal susceptibility testing of *Candida tropicalis*

**Figure 1:**
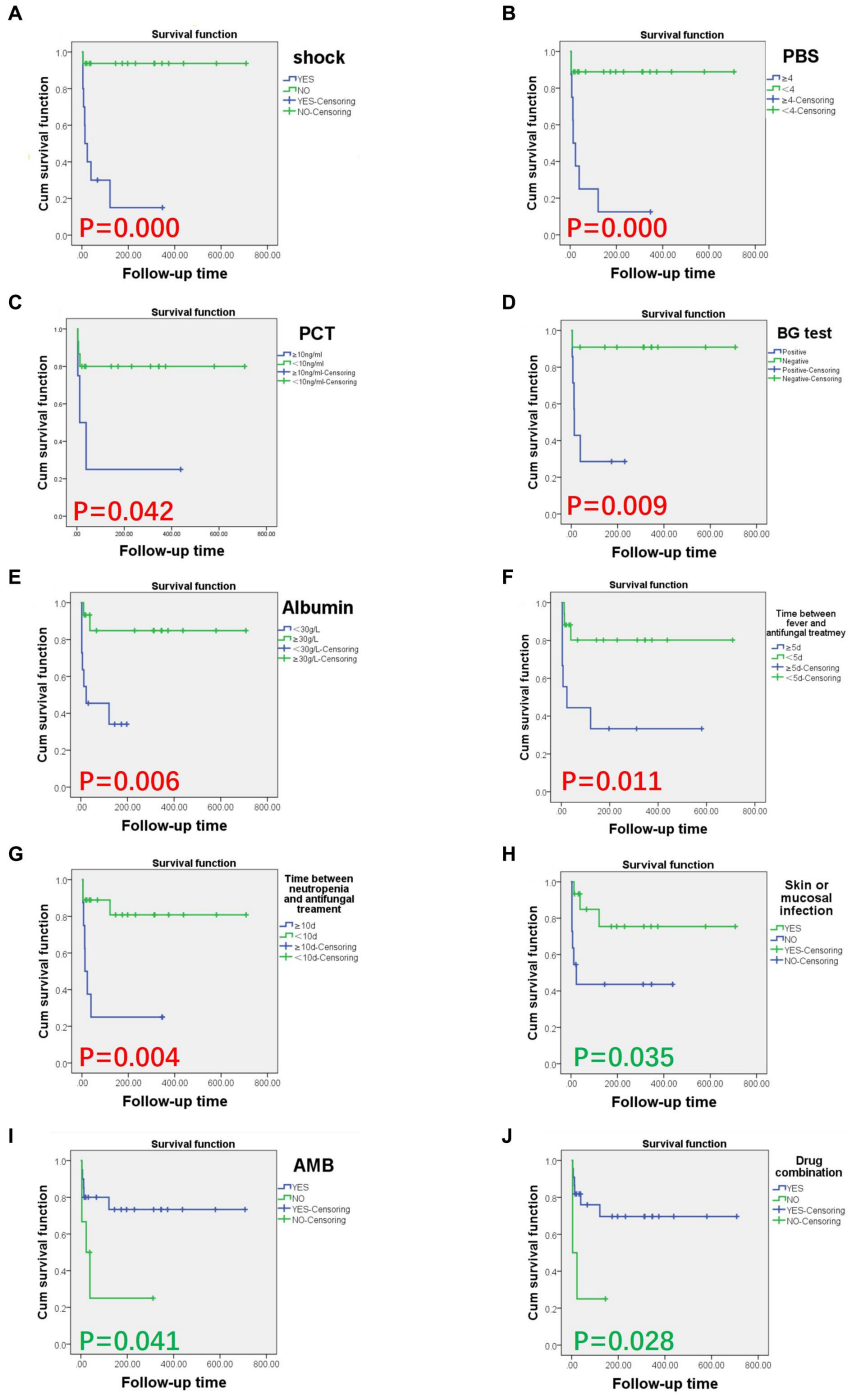
Survival curves of hematological disease patients with CTBI by risk and protective factors. Risk and protective factors significantly associated with CTBI-associated death (*p* < 0.05). Risk factors included **(A)** septic shock (*p* = 0.000), **(B)** Pitt bacteremia score ≥4 (*p* = 0.000), **(C)** PCT ≥ 10 ng/mL (*p* = 0.042), **(D)** BG ≥ 0.5 (*p* = 0.009), **(E)** serum albumin <30.0 g/L (*p* = 0.006), **(F)** time from fever to antifungal treatment ≥5 days (*p* = 0.011), and **(G)** time from neutropenia to antifungal treatment ≥10 days (*p* = 0.004). Protective factors included **(H)** skin or mucosal infection (*p* = 0.035), **(I)** treatment containing amphotericin B (*p* = 0.041), and **(J)** treatment involving a drug combination (*p* = 0.028). AMB, amphotericin B; BG, plasma (1,3)- β-D glucan assay; CTBI, *C. tropicalis* bloodstream infections; PBS, Pitt Bacteremia Score; PCT, procalcitonin.

Antifungal susceptibility testing was performed on 24 of the 26 *C. tropicalis* isolates (Table 5). In general, all isolates were sensitive to flucytosine and amphotericin B. The intermediate or resistance of *C. tropicalis* to fluconazole, itraconazole and voriconazole were 41.7, 50, and 41.7%, respectively. These findings indicated that *C. tropicalis* was highly resistant to azole drugs and sensitive to flucytosine and amphotericin B (Figure 1).

**Table 5:** Antifungal susceptibility testing of *C. tropicalis* isolates (*n* = 24).

Drug	Sensitive		Intermediate		Resistance	
Flucytosine	24	MIC ≤ 4 mg/L, *n* = 24	0		0	
Amphotericin B	24	MIC ≤ 0.5 mg/L, *n* = 24	0		0	
Fluconazole	14	MIC ≤ 1 mg/L, *n* = 14	2	MIC = 16 mg/L, *n* = 2	8	MIC = 64 mg/L, *n* = 1 MIC ≥ 128 mg/L, *n* = 7
Itraconazole	12	MIC ≤ 0.125 mg/L, *n* = 12	2	MIC = 0.25 mg/L, *n* = 2	9	MIC = 1 mg/L, *n* = 3 MIC = 2 mg/L, *n* = 4 MIC ≥ 4 mg/L, *n* = 2
Voriconazole	14	MIC ≤ 0.06 mg/L, *n* = 11 MIC = 0.125 mg/L, *n* = 3	1	MIC = 1 mg/L, *n* = 2	9	MIC = 2 mg/L, *n* = 1 MIC =4 mg/L, *n* = 3 MIC ≥8 mg/L, *n* = 5

MIC, minimal inhibitory concentration.

## 4. Discussion

*C. albicans* is the primary cause of CBI, however, *C. tropicalis* is becoming increasingly more common in patients with hematologic disorders and HSCT (Tan et al., 2015). This species is shown to have higher virulence than *C. albicans* at the time of neutropenia or mucosal infection (Deorukhkar et al., 2014). The current study found that all *C. tropicalis*-infected patients were neutropenic and more than half had skin or mucosal infections (57.7%), indicating a likely association between these factors and CTBI.

A prior study found that infection with *C. tropicalis* was more severe and had worse clinical outcomes, including a 30-day patient mortality rate of 44.1%, than non-albicans Candidemia (Ko et al., 2019). The current study identified a CTBI-attributable mortality rate of 34.6%, with clinical symptoms that included fever, respiratory symptoms, digestive tract symptoms, skin or mucosal infections, and septic shock. Septic shock, one of the most serious BSI complications, is more often associated with *C. tropicalis* than *C. albicans* infection (Zuza-Alves et al., 2017). In the current study, septic shock occurred in 38.5% of patients. This symptom was an independent risk factor for CTBI-attributable death (*p* = 0.006). These findings highlight how critical it is to actively prevent and control shock among patients with neutropenia and fever.

CTBI has no characteristic clinical manifestations and diagnosis is primarily dependent on blood culture results. However, blood culture positivity rates are low, and early or bacterial bloodstream infections can be difficult to identify, contributing to high mortality rates. Previous studies have shown that a PCT value ≤2 ng/mL in a critically ill septic patient is more likely the result of Candidemia than bacteremia (Martini et al., 2010). Indeed, the current study found that the PCT value of most patients (73.7%) was ≤2 ng/mL. The PCT value ≥4 ng/mL is a risk factor for CTBT-associated death among patients with hematologic malignancies (Chao Zhi et al., 2016). The present findings showed that a PCT value ≥10 ng/mL was linked to a poor prognosis (*p* = 0.042). These data indicate that a low PCT value is a more likely indicator of Candidemia; however, poor prognosis is most often associated with a significant rise in PCT values among patients with hemopathy and CTBI.

A large-scale, multicenter clinical study (Sun et al., 2015) found that risk factors of IFD among chemotherapy patients with malignant hematologic disease included neutropenia, AML or MDS, non-CR patients who receipt of induction chemotherapy or repeat induction chemotherapy, decreased serum albumin levels, and CVC. All patients in the current study were neutropenic, 61.5% had AML, and 42.3% had albumin levels <30.0 g/L, which correlated with a poor prognosis (*p* = 0.006). Thus, actively correcting hypoproteinemia can both prevent IFD and improve CTBI-associated outcomes.

The Pitt Bacteremia Score is used to measure acute illness severity and predict the mortality of patients with BSI (Al-Hasan and Baddour, 2020). This score was used as a stratification tool in pivotal multicenter studies of *Candida* spp.-related BSI (Nguyen, 1995). Patients with a Pitt Bacteremia Score < 4 were considered noncritically ill, while those with a Pitt Bacteremia Score ≥ 4 have a higher mortality risk and are classified as critically ill (Al-Hasan and Baddour, 2020). The current study determined the Pitt Bacteremia Score using the clinical symptoms of patients, identifying eight with a Pitt Bacteremia Score ≥ 4. Univariate analysis linked Pitt Bacteremia Score ≥ 4 to a poor prognosis (*p* = 0.001).

It is rarely study about the infection of *Candida* spp. with other pathogens. This study (Medina et al., 2020) documented the presence of mixed yeast infections. It showed that 6.5% of *C. tropicalis/C. glabrata* were infected in 122 cases, and All Candida isolates were susceptible to amphotericin B. In our study, mixed infection was no significant correlation with prognosis. We find a patient had *C. tropicalis* co-infected with *C. glabrata* and another had *C. tropicalis* co-infected with *C. albicans*, and they had a good prognosis in antifungal.

Azole-resistant *C. tropicalis* infections have markedly increased in recent years. Multiple studies have shown that almost half of Candidemia patients infected with *C. tropicalis* are azole-resistant (Chong et al., 2012; Chen et al., 2019; Arastehfar et al., 2020a,b). The current study found that 41.7, 50, and 41.7% of the 24 *C. tropicalis* isolates were resistant to fluconazole, itraconazole, or voriconazole, respectively. Our finding supports those studies. The seven *C. tropicalis*-infected patients changed treatments or added echinocandin, amphotericin B, and flucytosine to their current treatment because the original regimens, azole alone, were not effective. These results indicated that a therapeutic schedule containing amphotericin B and drug combinations protected hemopathy patients from CTBI-associated death (*p* = 0.041). Drug sensitivity testing also showed that *C. tropicalis* was sensitive to amphotericin B. In addition, echinocandin antifungal drugs have a strong bactericidal effect on *Candida* spp., and there are only a few reports of *C. tropicalis* resistance. Both Chinese and American guidelines (Pappas et al., 2015; Xiaojun, 2020) indicate that echinocandins are the preferred treatment for Candidemia in neutropenic patients, followed by amphotericin B and lipid formulation. In the current study, echinocandins had no significant effect on prognosis. This may be explained by the high cost of these drugs, which caused some patients to delay treatment initiation. Development of azole resistance in *C. tropicalis* may occur through increased levels of the cellular target, upregulation of genes controlling drug efflux, alterations in sterol synthesis and decreased affinity of azoles for the cellular target (Lupetti et al., 2002). The resistance may be related to the ERG11, ERG3, MDR1, and CDR1 genes (Gaur et al., 2008; Prasad and Goffeau, 2012; Forastiero et al., 2013; Branco et al., 2017; Zhou et al., 2018). It is likely that more mechanisms for Candida-specific drug resistance will be identified. Guidelines (Pappas et al., 2015; Xiaojun, 2020) emphasizes that these are important for the development of antifungal prophylaxis and empiric treatment. The current study confirmed that the timing of antifungal treatment initiation is equally important. A time between fever and antifungal treatment of ≥5 days and a time between agranulocytosis and antifungal treatment of ≥10 days correlated with a poor prognosis (*p* = 0.011 and *p* = 0004, respectively).

This study has several limitations. It is a single-centered retrospective analysis and the number of cases is relatively small, making it difficult to apply the findings to other settings. Multicenter and large-scale clinical studies are required to further understand CTBI-associated hematologic disorders. In addition, ongoing studies of *C. tropicalis*-specific virulence factors and resistance genes will help to inform new strategies for clinical diagnosis and treatment.

## 5. Conclusion

This study identified a high mortality rate among patients with CTBI-associated hematologic disorders, which the attributable mortality rate was 34.6%. Septic shock is an independent risk factor for death attributed to CTBI. There were poor prognoses in the time from fever to antifungal treatment initiation ≥5 days and the time between neutropenia and antifungal treatment ≥10 days, so early antifungal therapy can significantly reduce the risk of death. The intermediate or resistance of *C. tropicalis* to azole drugs was close to 50%, and it was sensitive to flucytosine and amphotericin B. According to our study, the preferred agent is amphotericin B and drug combinations should be considered for severe infections.

## Data availability statement

The raw data supporting the conclusions of this article will be made available by the authors, without undue reservation.

## Ethics statement

Written informed consent was obtained from the individual(s), and minor(s)’ legal guardian/next of kin, for the publication of any potentially identifiable images or data included in this article.

## Author contributions

WZ coordinated the study, initiated the project, and supervised. BY, ZW, and MW developed the protocol. BY and ZW analyzed the data and prepared the manuscript. WZ, MW, and YL critically revised the manuscript for important intellectual content. All authors contributed to the article and approved the submitted version.

## Funding

This work was supported by the China Postdoctoral Science Foundation (2020M673097).

## Conflict of interest

The authors declare that the research was conducted in the absence of any commercial or financial relationships that could be construed as a potential conflict of interest.

## Publisher’s note

All claims expressed in this article are solely those of the authors and do not necessarily represent those of their affiliated organizations, or those of the publisher, the editors and the reviewers. Any product that may be evaluated in this article, or claim that may be made by its manufacturer, is not guaranteed or endorsed by the publisher.
